# Association of the systemic immune-inflammation index with in-hospital malignant ventricular arrhythmia in patients with acute myocardial infarction

**DOI:** 10.3389/fmed.2026.1939873

**Published:** 2026-10-28

**Authors:** Zizhu Lian, Yao Li, Tianyi Zhang, Xiaohong Yang

**Affiliations:** 1 Department of Cardiovascular Surgery, The First Affiliated Hospital of Xi’an Jiaotong University, Xi’an, China; 2 Department of Emergency Medicine, The First Affiliated Hospital of Xi’an Jiaotong University, Xi’an, China; 3 Biobank, The First Affiliated Hospital of Xi’an Jiaotong University, Xi’an, China

**Keywords:** acute myocardial infarction, inflammation, malignant ventricular arrhythmia, risk stratification, systemic immune-inflammation index

## Abstract

**Background:**

Malignant ventricular arrhythmia (MVA) is a life-threatening in-hospital complication of acute myocardial infarction (AMI). The systemic immune-inflammation index (SII), derived from platelet, neutrophil, and lymphocyte counts, may reflect the inflammatory and thrombotic burden associated with arrhythmic vulnerability. This study investigated the association between SII and in-hospital MVA in patients with AMI.

**Methods:**

This retrospective study included 13,465 patients hospitalized for AMI at the First Affiliated Hospital of Xi'an Jiaotong University from January 2019 to December 2024. SII was calculated as platelet count × neutrophil count/lymphocyte count. Patients were grouped according to SII quartiles, and SII was also analyzed as standardized ln(SII). Logistic regression and restricted cubic spline analyses were used to assess the adjusted association and dose-response pattern, with subgroup and sensitivity analyses used to examine robustness. Separately, the apparent incremental performance of adding SII to a clinical model was assessed using the area under the receiver operating characteristic curve (AUC), net reclassification improvement (NRI), and integrated discrimination improvement (IDI).

**Results:**

During hospitalization, 440 patients (3.3%) developed MVA. MVA incidence increased from 1.37% in the lowest SII quartile (Q1) to 6.98% in the highest quartile (Q4). In the fully adjusted model, each 1-SD increase in ln(SII) was associated with higher odds of MVA (odds ratio [OR], 1.48; 95% confidence interval [CI], 1.34–1.64; *P* < 0.001). Compared with Q1, Q4 remained associated with MVA (OR, 2.91; 95% CI, 2.06–4.10; *P* < 0.001). The apparent AUC was 0.797 for the clinical model and 0.814 after adding standardized ln(SII) (*Δ*AUC = 0.017).

**Conclusion:**

Higher admission SII remained associated with greater odds of in-hospital MVA after multivariable adjustment. The incremental model performance was modest, and external validation is required before its predictive performance and clinical utility can be established.

## 1 Introduction

Acute myocardial infarction (AMI) remains a major cardiovascular emergency and continues to impose a substantial burden of early morbidity and mortality despite advances in reperfusion therapy, antithrombotic treatment, and intensive care management (1). Malignant ventricular arrhythmia (MVA), mainly sustained ventricular tachycardia and ventricular fibrillation, is among the most life-threatening in-hospital complications after AMI. It may precipitate hemodynamic collapse, necessitate emergent defibrillation, and ultimately result in sudden cardiac death (2). In the contemporary China Acute Myocardial Infarction Registry, sustained ventricular tachyarrhythmias occurred in 3.40% of hospitalized AMI patients, and any in-hospital arrhythmia was associated with markedly higher mortality than no arrhythmia (15.0% vs. 1.7%) (3). Post-AMI ventricular arrhythmogenesis involves not only acute ischemic electrical instability but also myocardial injury, inflammation, fibrosis, and adverse ventricular remodeling, highlighting the need for simple biomarkers to support early arrhythmic risk stratification (4).

Inflammation is involved throughout the course of AMI, from plaque rupture and coronary thrombosis to ischemia-reperfusion injury, myocardial necrosis, and subsequent cardiac repair (5). Pro-inflammatory cytokines, including interleukin−6, tumor necrosis factor-α, and interleukin-1β, may promote ventricular electrical instability by altering ion-channel function, calcium handling, action-potential duration, and repolarization reserve (6, 7). At the systemic level, this inflammatory-thrombotic response is partly reflected by circulating neutrophils, lymphocytes, and platelets. The systemic immune-inflammation index (SII), calculated as platelet count × neutrophil count/lymphocyte count, integrates these three routine blood-cell parameters and may therefore serve as a readily available composite marker of inflammation, immune imbalance, and platelet-related thrombosis after AMI (8).

Previous evidence on SII in coronary artery disease has mainly focused on prognosis rather than arrhythmic events. In patients with acute coronary syndrome (ACS), Su et al. reported that elevated SII was associated with increased mortality risk (9). In an AMI population, Wei et al. further showed that SII and related systemic inflammatory indices were associated with adverse cardiovascular outcomes and indicators of disease severity (10). More directly, Aksakal et al. found that SII was an independent predictor of in-hospital MVA in patients with ST-segment elevation myocardial infarction (11). In addition, a recent meta-analysis suggested that elevated SII was associated with an increased risk of major adverse cardiovascular events in patients undergoing percutaneous coronary intervention (PCI) (12). Nevertheless, evidence regarding SII and in-hospital MVA remains limited, particularly in broader hospitalized AMI cohorts including both ST-segment elevation myocardial infarction (STEMI) and non-ST-segment elevation myocardial infarction (NSTEMI) patients. Whether SII improves model performance beyond conventional clinical risk factors remains unclear. We hypothesized that higher admission SII would be associated with greater odds of in-hospital MVA. Therefore, this study investigated the association between SII and in-hospital MVA in a large cohort of hospitalized patients with AMI and evaluated the incremental performance of a clinical model after adding SII.

## 2 Materials and methods

### 2.1 Patient enrollment

This retrospective study included patients who were hospitalized for acute myocardial infarction (AMI) at the First Affiliated Hospital of Xi'an Jiaotong University from January 2019 to December 2024. AMI was diagnosed according to contemporary clinical guidelines using clinical symptoms, electrocardiographic findings, myocardial injury biomarkers, imaging examinations, and discharge diagnoses. Eligible patients were aged 18 years or older and had complete blood cell count data available for calculating the SII. Patients were excluded if they had prior myocardial infarction, sustained ventricular tachycardia/ventricular fibrillation, resuscitated cardiac arrest, or implantable cardioverter-defibrillator (ICD) implantation before the index hospitalization. Patients with autoimmune disease, severe infection, malignancy or hematologic disease, immunosuppressive therapy, or missing SII data were also excluded. These prespecified criteria were intended to define incident in-hospital MVA and reduce potential confounding from pre-existing arrhythmic conditions or conditions that could substantially affect blood cell counts. The patient screening, inclusion, and exclusion process is shown in Figure 1.

**Figure 1:**
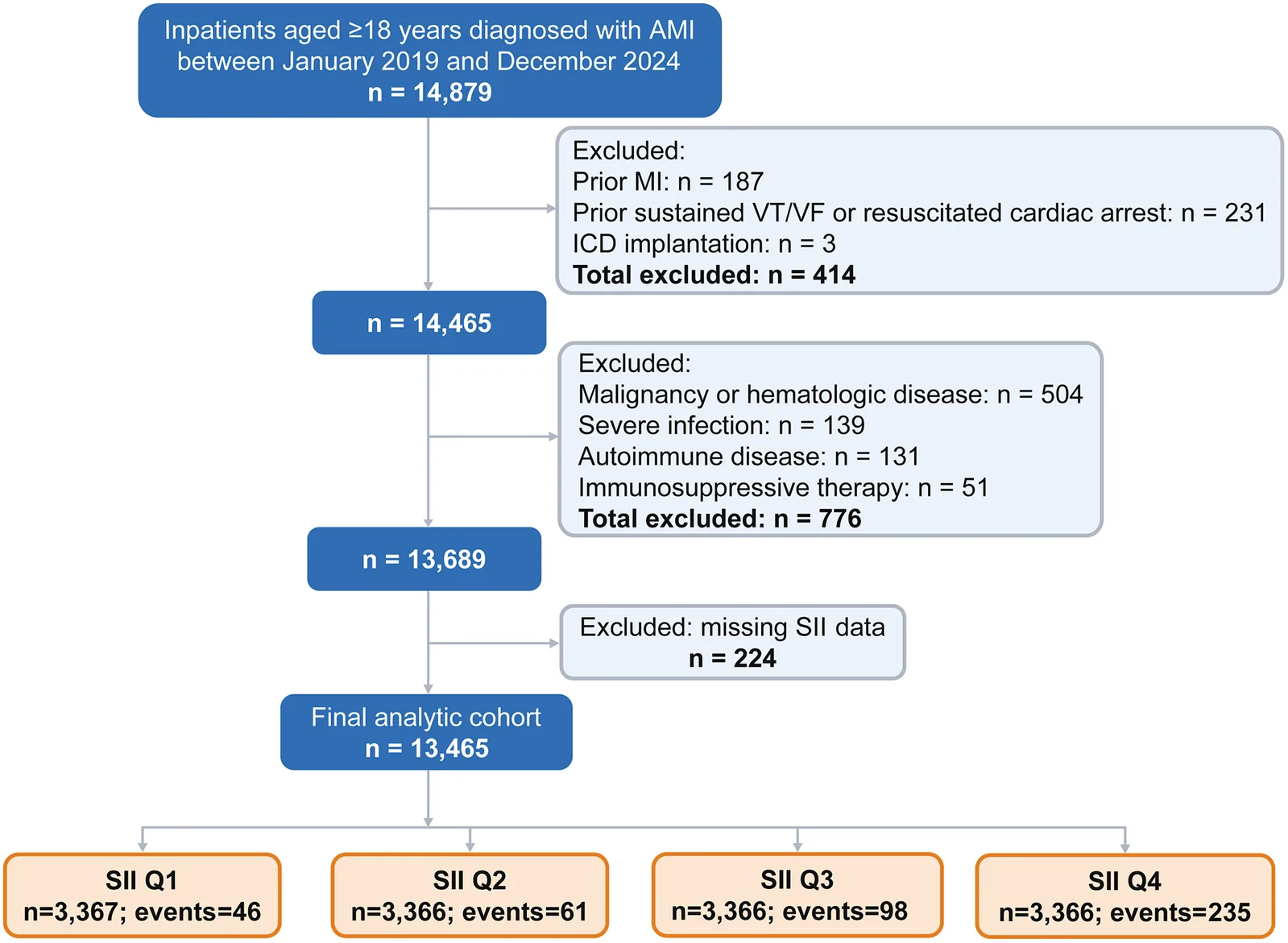
Study flowchart. Exclusion criteria were not mutually exclusive; totals represent unique patients. AMI, acute myocardial infarction; MVA, malignant ventricular arrhythmia; SII, systemic immune-inflammation index; MI, myocardial infarction; VT/VF, ventricular tachycardia/ventricular fibrillation; ICD, implantable cardioverter-defibrillator.

This study was approved by the Ethics Committee of the First Affiliated Hospital of Xi'an Jiaotong University (XJTU1AF2026LSKYY-0409) and was conducted in accordance with the Declaration of Helsinki. The requirement for informed consent was waived by the Ethics Committee because of the retrospective observational design.

### 2.2 Data collection and variable definitions

Demographic data, medical history, clinical presentation, laboratory results, treatment variables, and in-hospital outcomes were obtained from the Biobank of the First Affiliated Hospital of Xi’an Jiaotong University. The SII was calculated from the earliest available complete blood count during the index presentation, with all component cell counts measured using hospital laboratory analyzers, according to the following formula: platelet count × neutrophil count/lymphocyte count. The timing of the index blood sample was reviewed in relation to the first documented MVA event to verify temporal precedence. Patients were categorized by SII quartiles for descriptive and categorical regression analyses, whereas standardized ln(SII) was used for continuous regression and model-performance analyses.

The primary outcome was new-onset in-hospital MVA, initially identified from discharge diagnoses and subsequently reviewed against available contemporaneous medical records, including ECG or telemetry reports and documentation of resuscitation or defibrillation. To enhance the identification of clinically high-risk arrhythmic events, MVA was defined as ventricular fibrillation, sustained ventricular tachycardia, ventricular arrhythmia-related cardiac arrest or cardiopulmonary resuscitation, or defibrillation for ventricular arrhythmia. This definition focused on life-threatening ventricular arrhythmias and excluded transient or clinically uncertain rhythm abnormalities. Patients were classified into the MVA group and the control group according to whether MVA occurred during hospitalization.

### 2.3 Statistical analysis

Missing covariate data were handled using multiple imputation by chained equations (MICE), under a missing-at-random assumption conditional on the observed data. The imputation model included the outcome and available clinical and laboratory variables, with variables outside the primary analysis model serving as auxiliary predictors; patient identifiers were excluded, and the outcome was not imputed. Missingness among model covariates ranged from 0% to 6.39%, with variable-specific counts and proportions reported in Supplementary Table S1. Given the low overall missingness, five imputed datasets were generated using 10 iterations per dataset. Regression estimates were pooled using Rubin's rules. Predictive mean matching was used for continuous variables, and proportional odds logistic regression was used for ordered categorical variables. Continuous variables were presented as medians with interquartile ranges and compared using the Mann–Whitney *U*-test or Kruskal–Wallis test, as appropriate. Categorical variables were reported as counts and percentages and compared using the chi-square test or Fisher's exact test.

Logistic regression models were used to evaluate the association between SII and MVA. SII was analyzed as quartiles, per-quartile increase, and per 1-SD increase in ln-transformed SII. The hierarchical adjustment sets were constructed on clinical grounds, considering the relevance of covariates to AMI severity and ventricular arrhythmic risk, their potential relationship with systemic inflammation, their availability at presentation, and data completeness. Covariates were not selected by univariable *P*-value screening or automated stepwise procedures. Model 0 was unadjusted. Model 1 was adjusted for age and sex. Model 2 was further adjusted for Killip class entered as a categorical variable, heart rate, STEMI, anterior myocardial infarction, diabetes mellitus, atrial fibrillation, history of coronary artery disease, and history of stroke. Model 3 was additionally adjusted for low-density lipoprotein cholesterol (LDL-C), admission glucose, albumin, blood urea nitrogen, and ln-transformed glycated hemoglobin (HbA1c). Multicollinearity was assessed using variance inflation factors. Restricted cubic spline analysis was performed to assess the dose-response association between SII and MVA.

The incremental model performance after adding ln-transformed SII was assessed by comparing the clinical model with and without ln-transformed SII using the area under the receiver operating characteristic curve, paired DeLong test, calibration curves, decision curve analysis, category-free net reclassification improvement, and integrated discrimination improvement. The estimates presented in Table 3 and Figure 3 represent apparent performance in the study cohort. Optimism was estimated using 500 patient-level bootstrap samples, with identical resampling indices applied across the five imputed datasets. In each bootstrap sample, both models were refitted, patient-level predicted probabilities were averaged across imputations, and optimism-corrected AUC, calibration intercept, calibration slope, and Brier score were calculated. NRI, IDI, and decision-curve analyses were retained as secondary sample-internal assessments. Independent external validation was not performed. Exploratory apparent AUCs for the full-cohort clinical model and the model including ln(SII) were compared across prespecified age, AMI type, and PCI status strata using paired DeLong tests with Holm correction. Subgroup and interaction analyses were performed across five clinically relevant AMI phenotypes: AMI type, infarct location, Killip class, cardiogenic shock, and PCI status. Subgroup-specific models omitted the corresponding stratification variable. Interaction was tested in the full cohort by including the subgroup main effect and its product term with standardized ln(SII) in Model 3; Killip class was grouped as I, II, and III–IV. The five interaction *P* values were adjusted using the Holm method. Sensitivity analyses included a complete-case analysis; additional adjustment for ln(CK-MB) and ln(NT-proBNP); an expanded model that added ln(CK-MB), ln(NT-proBNP), ln(creatinine), hemoglobin, cardiogenic shock, and PCI status to the Model 3 covariates; restricted cohort analyses; analyses stratified by length of stay (≤3 days, >3 to 7 days, and >7 days), with interaction testing; and an alternative-outcome analysis restricted to documented ventricular fibrillation or sustained ventricular tachycardia. Statistical analyses were performed using R version 4.5.2. All tests were two-sided, and *P* < 0.05 was considered statistically significant.

## 3 Results

### 3.1 Study population and baseline characteristics

Among 13,465 included AMI patients, 440 (3.3%) developed in-hospital MVA. In all patients with MVA, the index blood sample used to calculate SII preceded the first documented MVA event. Among these patients, ventricular fibrillation was documented in 193 (43.9%), sustained ventricular tachycardia in 66 (15.0%), defibrillation in 213 (48.4%), and cardiac arrest or cardiopulmonary resuscitation in 179 (40.7%). Because multiple components could be documented during the same clinical event, these categories were not mutually exclusive. The remaining 115 cases (26.1%) were identified from MVA-related discharge diagnoses. Baseline characteristics according to SII quartiles are shown in Table 1.

**Table 1:** Baseline characteristics according to SII quartiles.

Variable	Overall (*n* = 13,465)	Q1 (*n* = 3,367)	Q2 (*n* = 3,366)	Q3 (*n* = 3,366)	Q4 (*n* = 3,366)	*P* value
Demographics						
Age, years	62.0 [53.0, 70.0]	63.0 [54.0, 70.0]	62.0 [53.0, 70.0]	61.0 [52.0, 69.0]	62.0 [52.0, 70.0]	0.006
Male sex, *n* (%)	10,786 (80.1)	2,727 (81.0)	2734 (81.2)	2698 (80.2)	2,627 (78.0)	0.004
Clinical presentation						
Length of stay, days	3.8 [2.3, 5.8]	3.8 [2.3, 5.8]	3.8 [2.3, 5.8]	3.8 [2.3, 5.8]	3.6 [2.4, 5.8]	0.798
ST-segment elevation myocardial infarction, *n* (%)	2,175 (16.2)	385 (11.4)	448 (13.3)	573 (17.0)	769 (22.8)	<0.001
Anterior myocardial infarction, *n* (%)	4,733 (35.2)	936 (27.8)	1,072 (31.8)	1,220 (36.2)	1,505 (44.7)	<0.001
Killip class, *n* (%)						<0.001
I	9,637 (71.6)	2,536 (75.3)	2,515 (74.7)	2,473 (73.5)	2,113 (62.8)	
II	2,857 (21.2)	706 (21.0)	691 (20.5)	702 (20.9)	758 (22.5)	
III	444 (3.3)	68 (2.0)	82 (2.4)	99 (2.9)	195 (5.8)	
IV	527 (3.9)	57 (1.7)	78 (2.3)	92 (2.7)	300 (8.9)	
Cardiogenic shock, *n* (%)	254 (1.9)	24 (0.7)	37 (1.1)	43 (1.3)	150 (4.5)	<0.001
Heart rate, beats/min	77 [68, 88]	73 [65, 82]	76 [67, 85]	78 [69, 88]	82 [71, 95]	<0.001
Body temperature, °C	36.3 [36.0, 36.5]	36.3 [36.0, 36.5]	36.3 [36.1, 36.5]	36.3 [36.1, 36.5]	36.3 [36.0, 36.5]	0.033
Medical history						
Hypertension, *n* (%)	7,572 (56.2)	1,848 (54.9)	1,990 (59.1)	1,891 (56.2)	1,843 (54.8)	<0.001
Diabetes mellitus, *n* (%)	4,407 (32.7)	1,131 (33.6)	1,142 (33.9)	1,119 (33.2)	1,015 (30.2)	0.003
Hyperlipidemia, *n* (%)	1,736 (12.9)	443 (13.2)	443 (13.2)	423 (12.6)	427 (12.7)	0.832
History of coronary artery disease, *n* (%)	1,767 (13.1)	523 (15.5)	460 (13.7)	404 (12.0)	380 (11.3)	<0.001
Prior PCI or coronary artery bypass grafting (CABG), *n* (%)	908 (6.7)	262 (7.8)	238 (7.1)	215 (6.4)	193 (5.7)	0.006
Chronic kidney disease, *n* (%)	1,251 (9.3)	275 (8.2)	322 (9.6)	309 (9.2)	345 (10.2)	0.028
Atrial fibrillation, *n* (%)	788 (5.9)	167 (5.0)	171 (5.1)	178 (5.3)	272 (8.1)	<0.001
History of stroke, *n* (%)	2,167 (16.1)	604 (17.9)	559 (16.6)	542 (16.1)	462 (13.7)	<0.001
Smoking history, *n* (%)	7,539 (56.0)	1,985 (59.0)	1,911 (56.8)	1,886 (56.0)	1,757 (52.2)	<0.001
Laboratory findings						
White blood cell count, ×10⁹/L	8.37 [6.49, 10.93]	6.35 [5.27, 7.77]	7.57 [6.26, 9.25]	9.02 [7.35, 11.02]	11.75 [9.63, 14.51]	<0.001
Neutrophil count, ×10⁹/L	6.16 [4.45, 8.77]	3.97 [3.20, 4.93]	5.36 [4.38, 6.64]	7.03 [5.62, 8.75]	10.10 [8.22, 12.69]	<0.001
Lymphocyte count, ×10⁹/L	1.38 [1.02, 1.84]	1.77 [1.38, 2.26]	1.54 [1.20, 1.96]	1.32 [1.04, 1.69]	0.96 [0.72, 1.26]	<0.001
Platelet count, ×10⁹/L	208.0 [170.0, 253.0]	179.0 [145.0, 215.0]	204.0 [169.0, 244.0]	218.0 [181.0, 262.0]	236.0 [194.0, 282.0]	<0.001
Hemoglobin, g/L	140.0 [127.0, 152.0]	139.0 [128.0, 150.0]	140.0 [128.0, 151.0]	141.0 [127.0, 153.0]	141.0 [126.0, 153.0]	0.072
Systemic immune-inflammation index	900.0 [561.8, 1,542.2]	417.0 [330.0, 491.9]	715.2 [634.6, 802.5]	1,149.7 [1,015.2, 1,313.4]	2,292.2 [1,826.7, 3,180.3]	<0.001
Creatine kinase-MB, U/L	24.4 [14.0, 75.8]	16.6 [12.0, 30.4]	19.8 [13.0, 49.3]	31.0 [15.3, 93.0]	58.2 [22.0, 168.9]	<0.001
Creatine kinase, U/L	209.0 [89.0, 716.0]	120.0 [74.0, 276.0]	153.0 [77.0, 487.5]	287.0 [98.0, 895.8]	544.0 [169.0, 1,442.5]	<0.001
Lactate dehydrogenase, U/L	275.0 [217.0, 411.0]	233.0 [199.0, 301.0]	259.0 [210.0, 358.8]	293.0 [228.0, 446.8]	351.0 [254.0, 602.5]	<0.001
NT-proBNP, pg/mL	660.0 [213.0, 1,931.0]	496.0 [182.6, 1,291.0]	664.8 [242.2, 1,800.2]	742.0 [246.0, 2,108.0]	830.0 [191.3, 3,151.8]	<0.001
Creatinine, μmol/L	66.0 [55.0, 80.0]	66.0 [56.0, 78.0]	66.0 [55.0, 79.0]	65.0 [54.0, 80.0]	66.0 [54.0, 83.0]	0.182
Blood urea nitrogen, mmol/L	5.55 [4.47, 6.98]	5.43 [4.46, 6.70]	5.45 [4.38, 6.81]	5.47 [4.38, 6.93]	5.87 [4.67, 7.59]	<0.001
Admission glucose, mmol/L	6.54 [5.32, 8.73]	6.14 [5.10, 8.14]	6.32 [5.20, 8.50]	6.56 [5.37, 8.61]	7.19 [5.83, 9.63]	<0.001
Hemoglobin A1c, %	6.00 [5.60, 7.00]	6.00 [5.60, 7.00]	6.00 [5.70, 7.00]	6.00 [5.60, 7.00]	6.00 [5.60, 6.80]	<0.001
Triglycerides, mmol/L	1.31 [0.94, 1.87]	1.33 [0.97, 1.88]	1.36 [0.99, 1.93]	1.31 [0.94, 1.85]	1.25 [0.87, 1.80]	<0.001
Low-density lipoprotein cholesterol, mmol/L	2.29 [1.76, 2.90]	2.21 [1.70, 2.82]	2.23 [1.74, 2.85]	2.32 [1.77, 2.90]	2.39 [1.84, 3.01]	<0.001
High-density lipoprotein cholesterol, mmol/L	0.92 [0.78, 1.07]	0.90 [0.77, 1.05]	0.90 [0.77, 1.05]	0.92 [0.78, 1.06]	0.96 [0.81, 1.12]	<0.001
Albumin, g/L	38.60 [35.20, 41.90]	38.80 [35.70, 41.90]	38.70 [35.30, 41.70]	38.70 [35.10, 42.00]	38.50 [34.70, 42.10]	0.013
D-dimer, mg/L	0.57 [0.36, 0.95]	0.51 [0.32, 0.73]	0.55 [0.33, 0.82]	0.57 [0.35, 0.92]	0.76 [0.42, 1.70]	<0.001
Treatment						
Percutaneous coronary intervention, *n* (%)	9,690 (72.0)	2,458 (73.0)	2,466 (73.3)	2,422 (72.0)	2,344 (69.6)	0.003

Values are presented as median [interquartile range] or *n* (%). *P* values compare groups across SII quartiles.

SII, systemic immune-inflammation index; Q, quartile; STEMI, ST-segment elevation myocardial infarction; PCI, percutaneous coronary intervention; CABG, coronary artery bypass grafting; CK-MB, creatine kinase-MB; NT-proBNP, N-terminal pro-B-type natriuretic peptide.

Compared with the lowest quartile, patients in the highest SII quartile presented with a more severe AMI phenotype, including higher proportions of STEMI, anterior myocardial infarction, Killip class III or IV, and cardiogenic shock, together with a higher admission heart rate. They also showed a stronger inflammatory and hematologic response, characterized by higher white blood cell, neutrophil, and platelet counts and lower lymphocyte counts. Markers of myocardial injury and metabolic stress, including creatine kinase-MB (CK-MB), creatine kinase, lactate dehydrogenase, admission glucose, and D-dimer, were also higher in the highest SII quartile.

### 3.2 Association between SII and MVA

The incidence of MVA increased progressively across SII quartiles, from 1.37% in Q1 to 6.98% in Q4 (Q1: 46/3367; Q2: 61/3366; Q3: 98/3366; Q4: 235/3366) (Figure 2A). The association between SII and MVA remained after multivariable adjustment (Table 2). In the fully adjusted model, each 1-SD increase in ln(SII) corresponded to higher odds of MVA (odds ratio [OR], 1.48; 95% confidence interval [CI], 1.34–1.64; *P* < 0.001). Patients in the highest SII quartile had nearly threefold higher odds of MVA than those in the lowest quartile (OR, 2.91; 95% CI, 2.06–4.10; *P* < 0.001). A graded increase in MVA risk was also observed across SII quartiles (per-quartile increase: OR, 1.45; 95% CI, 1.31–1.61; *P* < 0.001).

**Figure 2:**
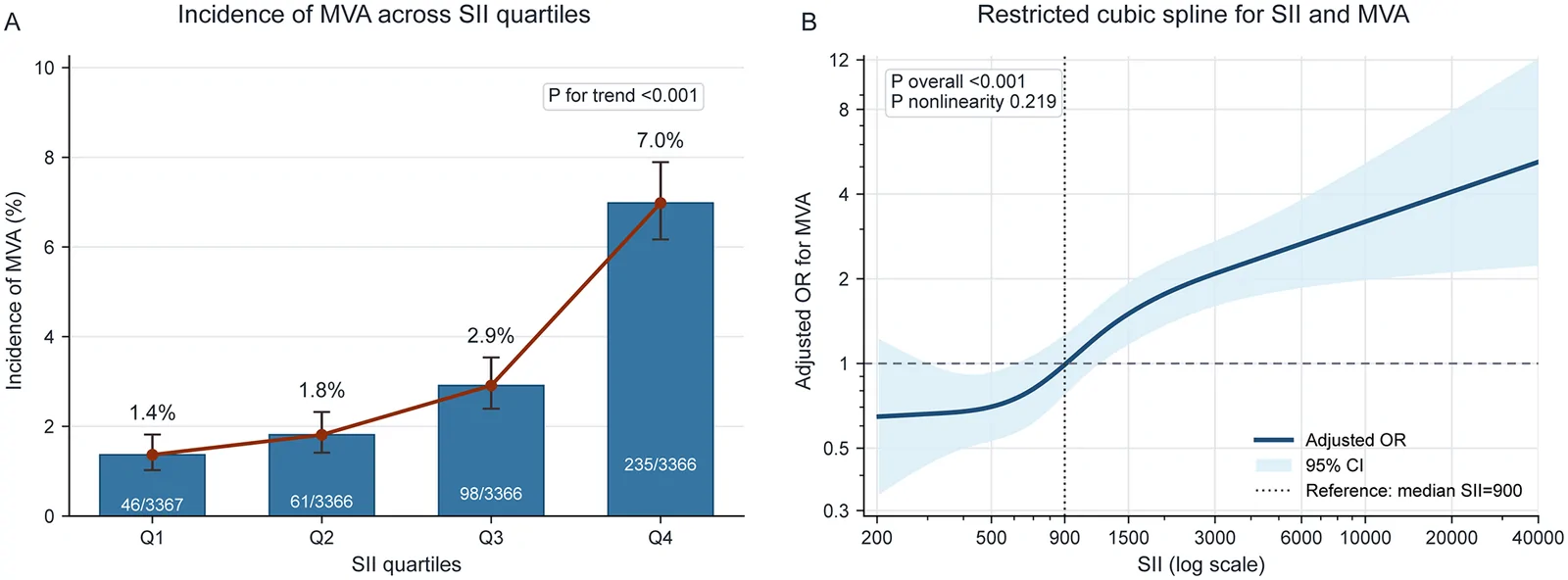
MVA incidence and dose–response pattern across SII levels. **(A)** Incidence of in-hospital MVA by SII quartile. **(B)** Restricted cubic spline for the association between SII and MVA; the model was fully adjusted. Quartile cutoffs were 561.8, 900.0, and 1542.2. SII, systemic immune-inflammation index; MVA, malignant ventricular arrhythmia; Q, quartile; OR, odds ratio; CI, confidence interval.

**Table 2:** Association between SII and MVA.

Category/unit	Events/N (%)	Model 0		Model 1		Model 2		Model 3	
		OR (95% CI)	*P* value	OR (95% CI)	*P* value	OR (95% CI)	*P* value	OR (95% CI)	*P* value
Q1	46/3,367 (1.4)	Reference		Reference		Reference		Reference	
Q2	61/3,366 (1.8)	1.33 (0.91–1.96)	0.145	1.34 (0.91–1.97)	0.139	1.23 (0.83–1.82)	0.302	1.27 (0.85–1.88)	0.239
Q3	98/3,366 (2.9)	2.16 (1.52–3.08)	<0.001	2.19 (1.53–3.11)	<0.001	1.88 (1.31–2.70)	<0.001	1.88 (1.31–2.72)	<0.001
Q4	235/3,366 (7.0)	5.42 (3.94–7.46)	<0.001	5.47 (3.97–7.53)	<0.001	2.95 (2.10–4.14)	<0.001	2.91 (2.06–4.10)	<0.001
Per-quartile increase		1.87 (1.69–2.06)	<0.001	1.87 (1.70–2.07)	<0.001	1.47 (1.33–1.63)	<0.001	1.45 (1.31–1.61)	<0.001
Per 1-SD increase in ln(SII)	440/13,465 (3.3)	2.00 (1.83–2.19)	<0.001	2.01 (1.84–2.20)	<0.001	1.50 (1.36–1.66)	<0.001	1.48 (1.34–1.64)	<0.001

Model 0 was unadjusted.

Model 1 was adjusted for age and sex.

Model 2 was further adjusted for Killip class (categorical), heart rate, STEMI, anterior myocardial infarction, diabetes mellitus, atrial fibrillation, history of coronary artery disease, and history of stroke.

Model 3 was further adjusted for LDL-C, admission glucose, albumin, blood urea nitrogen, and ln(HbA1c).

SII, systemic immune-inflammation index; MVA, malignant ventricular arrhythmia; Q, quartile; SD, standard deviation; OR, odds ratio; CI, confidence interval; STEMI, ST-segment elevation myocardial infarction; LDL-C, low-density lipoprotein cholesterol; HbA1c, glycated hemoglobin.

Restricted cubic spline analysis confirmed an overall positive association between SII and MVA (*P* overall < 0.001), without evidence of nonlinearity (*P* for nonlinearity = 0.218) (Figure 2B). Multicollinearity diagnostics showed no evidence of problematic collinearity in the fully adjusted model, with VIFs ranging from 1.03 to 2.64; the VIF for ln(SII) was 1.15.

### 3.3 Incremental model performance after adding SII

Table 3 and Figure 3 summarize the changes in model performance after adding SII. The clinical model had an area under the receiver operating characteristic curve (AUC) of 0.797 (95% CI, 0.774–0.820). After adding ln(SII), the AUC increased to 0.814 (95% CI, 0.791–0.837), with a *Δ*AUC of 0.017 (*P* = 0.002).

**Table 3:** Incremental model performance after adding SII to the clinical model.

Model	AUC	95% CI	*Δ*AUC	*P* for *Δ*AUC	NRI (95% CI)	*P* value	IDI (95% CI)	*P* value
Clinical model	0.797	0.774–0.820			Reference	–	Reference	–
Clinical model + ln(SII)	0.814	0.791–0.837	0.017	0.002	0.370 (0.277–0.466)	<0.001	0.009 (0.003–0.014)	<0.001

The incremental model additionally included standardized ln(SII). SII, systemic immune-inflammation index; AUC, area under the receiver operating characteristic curve; CI, confidence interval; NRI, net reclassification improvement; IDI, integrated discrimination improvement. Values are apparent estimates from the study cohort; optimism-corrected internal-validation results are reported in Supplementary Table S2.

**Figure 3:**
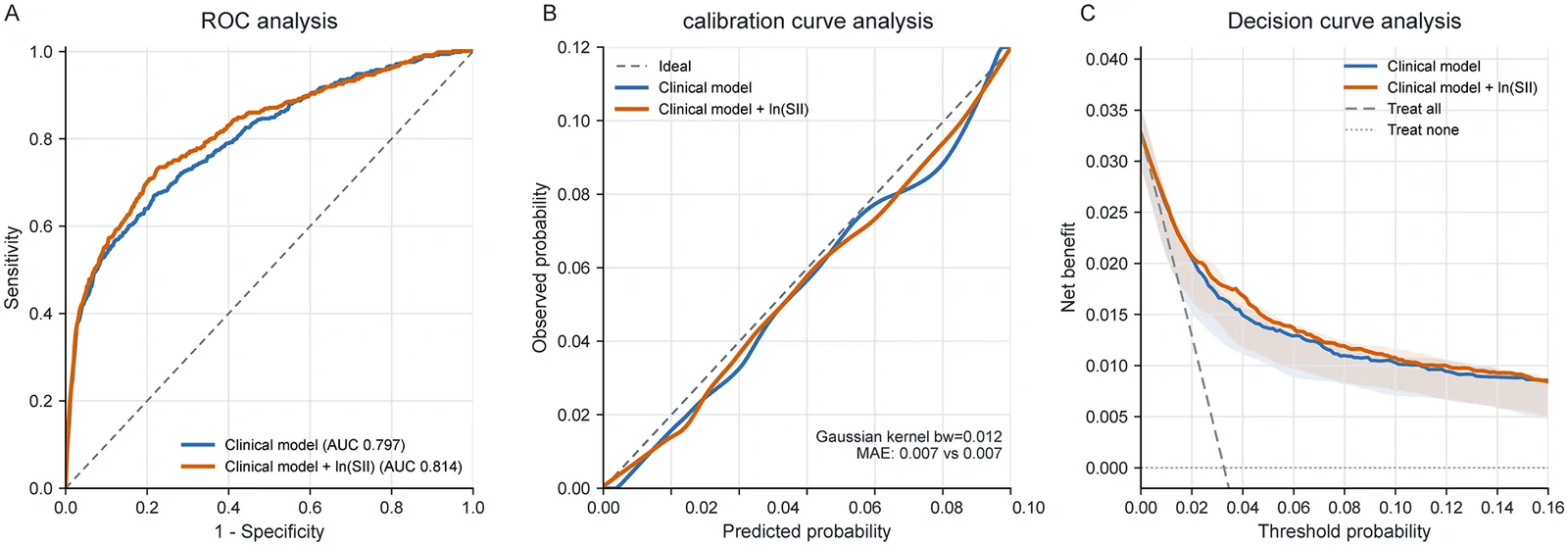
Incremental model performance after adding SII to the clinical model. **(A)** Receiver operating characteristic curves. **(B)** Calibration curves. **(C)** Decision curve analysis. The clinical model included the final 15 covariates, and the incremental model additionally included standardized ln(SII). SII, systemic immune-inflammation index; ROC, receiver operating characteristic; AUC, area under the curve; DCA, decision curve analysis.

Adding ln(SII) also improved risk reclassification, as reflected by a category-free net reclassification improvement of 0.370 (95% CI, 0.277–0.466; *P* < 0.001) and an integrated discrimination improvement of 0.009 (95% CI, 0.003–0.014; *P* < 0.001). Apparent calibration in the study cohort was acceptable, and decision curve analysis showed a slightly higher net benefit after adding SII across the evaluated range of threshold probabilities. Bootstrap internal validation yielded optimism-corrected AUCs of 0.788 and 0.806 for the clinical model and the model including ln(SII), respectively, with an optimism-corrected *Δ*AUC of 0.018. The corresponding optimism-corrected calibration slopes were 0.983 and 0.981, and the Brier scores were 0.0286 and 0.0283, respectively (Supplementary Table S2). Apparent incremental discrimination after adding ln(SII) was not uniform across age, AMI type, and PCI status strata (Supplementary Table S3).

### 3.4 Effect modification by AMI phenotype

Figure 4 shows phenotype-stratified analyses. After Holm correction, evidence of interaction remained for AMI type, Killip class, and PCI status (adjusted *P* = 0.002, 0.044, and 0.004, respectively). The association between SII and MVA was stronger in patients with NSTEMI than in those with STEMI, attenuated across increasing Killip classes, and stronger in patients undergoing PCI than in those not undergoing PCI. No evidence of interaction was observed for infarct location or cardiogenic shock. Given the multiple comparisons and limited event numbers in some strata, these findings should be considered exploratory.

**Figure 4:**
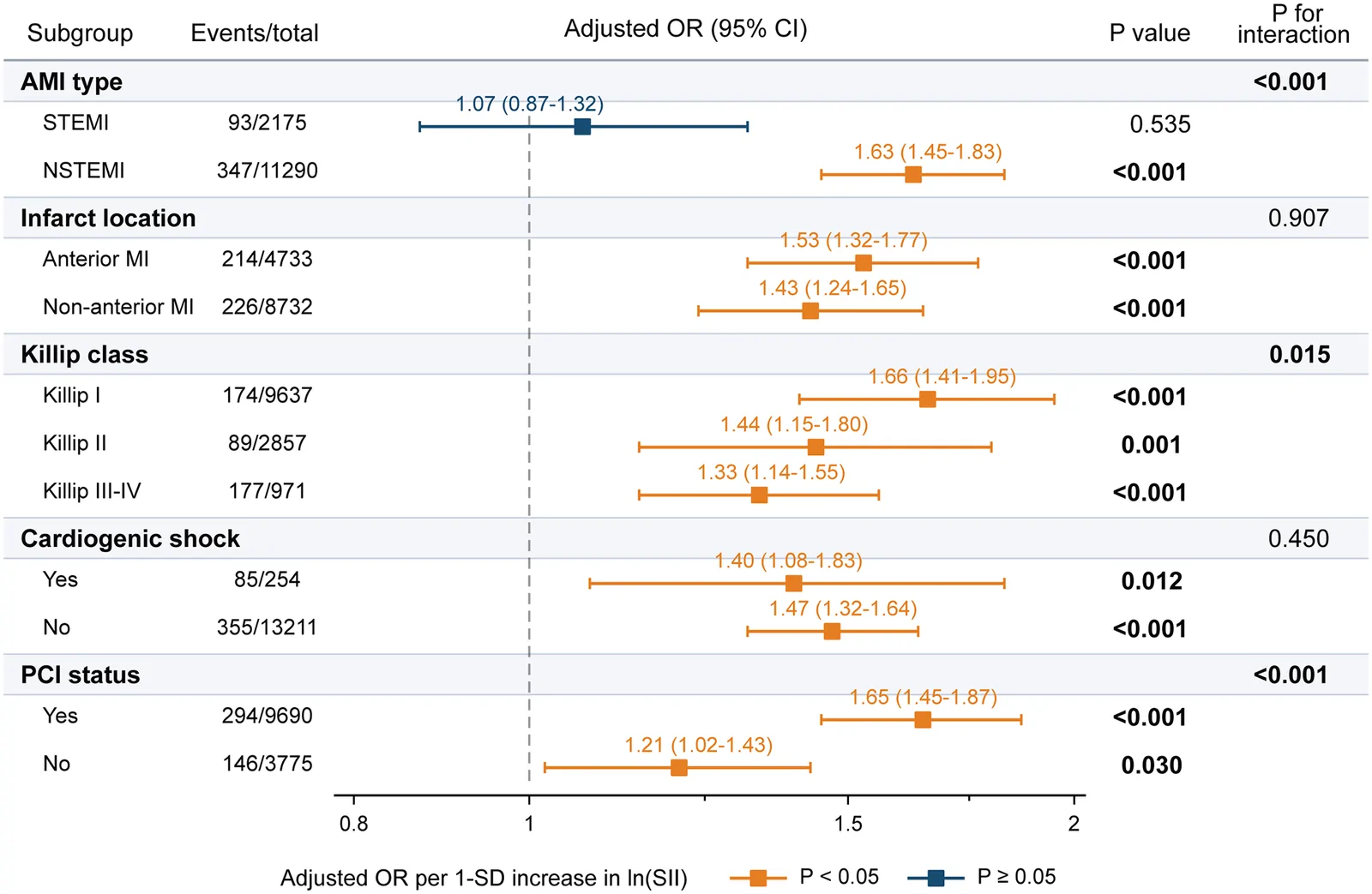
Phenotype-specific association between SII and MVA in AMI. Odds ratios were estimated per 1-SD increase in ln(SII). Subgroup models were adjusted for prespecified clinical covariates, with the corresponding stratification variable omitted. *P* for interaction was calculated using multiplicative interaction terms. Nominal interaction *P* values are shown; Holm-adjusted results are reported in the text. SII, systemic immune-inflammation index; MVA, malignant ventricular arrhythmia; AMI, acute myocardial infarction; SD, standard deviation; OR, odds ratio; CI, confidence interval; STEMI, ST-segment elevation myocardial infarction; NSTEMI, non-ST-segment elevation myocardial infarction; PCI, percutaneous coronary intervention.

### 3.5 Sensitivity analyses

Sensitivity analyses included a complete-case analysis using complete cases from the original non-imputed dataset (Supplementary Table S4), additional adjustment for creatine kinase-MB and N-terminal pro-B-type natriuretic peptide (Supplementary Table S5), and restricted analyses after excluding patients with cardiogenic shock (Supplementary Table S6), chronic kidney disease (Supplementary Table S7), or Killip class IV (Supplementary Table S8). In the complete-case analysis, ln(SII) remained associated with MVA after full adjustment (OR per 1-SD increase, 1.47; 95% CI, 1.31–1.65; *P* < 0.001), and the highest SII quartile was still associated with a higher risk of MVA compared with the lowest quartile (Q4 vs. Q1: OR, 3.04; 95% CI, 2.04–4.54; *P* < 0.001). After additional adjustment for creatine kinase-MB and N-terminal pro-B-type natriuretic peptide, the association was slightly attenuated but remained significant (OR per 1-SD increase in ln(SII), 1.44; 95% CI, 1.30–1.60; *P* < 0.001; Q4 vs. Q1: OR, 2.74; 95% CI, 1.91–3.92; *P* < 0.001). The association remained similar in the expanded sensitivity model (OR per 1-SD increase in ln(SII), 1.47; 95% CI, 1.32–1.63; *P* < 0.001).

The results were also consistent after excluding patients with cardiogenic shock, chronic kidney disease, or Killip class IV. In these restricted analyses, the Model 3 ORs per 1-SD increase in ln(SII) were 1.47 (95% CI, 1.32–1.64), 1.54 (95% CI, 1.38–1.72), and 1.56 (95% CI, 1.38–1.76), respectively. The corresponding ORs for Q4 vs. Q1 were 2.87 (95% CI, 2.00–4.13), 3.22 (95% CI, 2.22–4.67), and 3.40 (95% CI, 2.27–5.08), respectively. Overall, the sensitivity analyses supported the robustness of the SII-MVA association across different missing-data handling strategies, additional biomarker adjustment, and clinically relevant cohort restrictions. When stratified by length of stay, the adjusted ORs per 1-SD increase in ln-transformed SII were 1.69 (95% CI, 1.42–2.01), 1.55 (95% CI, 1.32–1.81), and 1.17 (95% CI, 0.94–1.46) for stays of ≤3 days, >3 to 7 days, and >7 days, respectively (*P* for interaction = 0.001; Supplementary Table S9). When the outcome was restricted to documented ventricular fibrillation or sustained ventricular tachycardia (224 events), the association remained after full adjustment (OR per 1-SD increase in ln(SII), 1.69; 95% CI, 1.47–1.93; *P* < 0.001; Supplementary Table S10).

## 4 Discussion

In this large retrospective cohort of 13,465 hospitalized patients with AMI, 440 patients (3.3%) developed in-hospital MVA. Higher SII remained associated with increased odds of MVA after multivariable adjustment, with each 1-SD increase in ln(SII) associated with 48% higher odds of MVA and the highest SII quartile showing nearly threefold higher odds than the lowest quartile. The incidence of MVA increased progressively across SII quartiles from 1.37% to 6.98%, and adding ln(SII) to the clinical model produced a modest apparent improvement in discrimination. Exploratory subgroup analyses suggested possible heterogeneity in the SII–MVA association by AMI type, Killip class, and PCI status.

Our findings partly overlap with, but also extend, prior evidence on inflammation-based prediction after AMI. Previous meta-analytic evidence has shown that elevated SII is associated with adverse outcomes in AMI, including major adverse cardiovascular events, contrast-induced nephropathy, and mortality (13). More specifically, Aksakal et al. reported that SII independently predicted in-hospital MVA in patients with STEMI (11). More recently, Han et al. developed interpretable machine-learning models using systemic inflammation indices to predict in-hospital MVA in STEMI patients undergoing emergency PCI (14). These studies support a link between systemic inflammation and early malignant arrhythmic risk, but they were largely restricted to STEMI or emergency PCI populations. Recent evidence has extended this literature to NSTEMI. Kafalı et al. compared SII with the systemic inflammatory response index and found that only SII remained independently associated with in-hospital mortality after adjustment in patients with NSTEMI (15). In a broader ACS cohort, Babes et al. reported significant differences in SII across clinical subtypes, with the highest levels in STEMI, intermediate levels in NSTEMI, and the lowest levels in unstable angina (16). Against this background, our study included a broader hospitalized AMI cohort and showed that the association between SII and MVA differed across AMI phenotypes. Significant effect modification was observed by AMI type, Killip class, and PCI status. Although SII levels were highest in STEMI in the previous ACS comparison, the SII–MVA association in our cohort was stronger in NSTEMI, suggesting that phenotype-related differences in inflammatory burden may not parallel the strength of association with a specific outcome. This pattern may reflect differences in patient characteristics, reperfusion status, arrhythmia definition, or the relative contributions of acute ischemic instability and systemic inflammation. The PCI-related finding was more consistent with previous evidence from STEMI patients undergoing emergency PCI, as the association was stronger in PCI-treated patients. For Killip class, directly comparable SII-MVA evidence is limited, as previous studies generally used Killip class as a severity marker or adjustment covariate. These subgroup findings are exploratory and may be influenced by residual confounding and treatment-selection bias.

The observed association between elevated SII and MVA after AMI can be biologically contextualized by the integrated cellular components of SII. However, because SII was also associated with multiple markers of AMI severity, it may partly reflect overall acute disease burden rather than a distinct inflammatory arrhythmogenic mechanism. Because SII combines neutrophil, platelet, and lymphocyte counts, a higher value may reflect a coordinated inflammatory-thrombotic state characterized by neutrophil and platelet predominance with relative lymphopenia. After AMI, inflammation is required for necrotic tissue clearance, infarct repair, extracellular matrix turnover, and ventricular remodeling; however, excessive or dysregulated inflammation may aggravate myocardial injury and promote electrical instability (17, 18). Neutrophils, as early responders to ischemic myocardium, can amplify tissue injury through reactive oxygen species generation, protease release, cytokine signaling, and formation of neutrophil extracellular traps (NETs) (19). Experimental evidence further suggests that neutrophil-driven immune activation may contribute to post-infarction electrical instability, whereas macrophage-mediated clearance may counteract electrical storm, indicating that the balance of immune-cell responses may influence arrhythmic susceptibility (20). NETs may connect inflammation with thrombosis by promoting endothelial injury, platelet activation, fibrin deposition, microvascular obstruction, and no-reflow during ischemia-reperfusion injury (21, 22). In parallel, platelet activation may intensify immunothrombosis through platelet-neutrophil aggregates, cytokine release, distal embolization, and microvascular thrombosis (23). At the electrophysiological level, inflammatory mediators may alter ion-channel function, calcium handling, connexin expression, autonomic tone, and cardiomyocyte-macrophage interactions, thereby lowering the threshold for triggered activity or reentry (24, 25). Coronary microvascular obstruction after AMI is associated with larger infarct size, impaired ventricular function, and adverse remodeling, providing a plausible substrate for ventricular arrhythmias (26). Relative lymphopenia may reflect neuroendocrine stress and impaired resolution of inflammation, and studies of inflammatory ratios after myocardial infarction support the prognostic relevance of neutrophil-dominant and lymphocyte-depleted immune profiles (27). Taken together, elevated SII may represent a peripheral signature of neutrophil-driven injury, platelet-mediated immunothrombosis, microvascular dysfunction, and insufficient immune regulation. These interconnected processes provide a biologically plausible framework linking high SII with ischemic heterogeneity, repolarization impairment, and early ventricular remodeling after AMI.

The subgroup findings further suggest that the association between SII and MVA may differ across AMI phenotypes. The stronger association observed in NSTEMI than in STEMI may partly reflect differences in the predominant determinants of ventricular arrhythmias. In STEMI, early ventricular arrhythmias are often strongly influenced by abrupt coronary occlusion, transmural ischemia, and reperfusion-related electrical instability, which may reduce the relative contribution of systemic inflammation to arrhythmic risk (28, 29). In NSTEMI, persistent thrombotic activity, endothelial dysfunction, and microvascular impairment may allow the inflammatory-thrombotic state reflected by SII to remain more relevant to arrhythmic vulnerability (30, 31). The attenuation of the association across higher Killip classes may similarly reflect the increasing influence of myocardial injury, heart failure, and hemodynamic instability, whereas SII may capture residual risk not fully reflected by bedside severity assessment in patients with Killip class I. The stronger association among PCI-treated patients may be related to reperfusion-associated inflammatory and thrombotic responses; however, PCI status may also reflect treatment selection and differences in the underlying clinical condition (32, 33). These interpretations provide possible clinical context rather than evidence of distinct biological mechanisms, and the subgroup findings should remain exploratory given the potential for residual confounding, multiple testing, and limited event numbers in some strata.

These findings may inform further evaluation of SII in early risk assessment. SII is derived from routine complete blood count parameters and can be obtained at admission without additional cost. Adding SII produced a modest apparent improvement in discrimination (*Δ*AUC, 0.017), which remained similar after bootstrap optimism correction (corrected *Δ*AUC, 0.018). The NRI, IDI, and decision-curve findings were directionally consistent but remain secondary, sample-internal analyses. These results suggest complementary prognostic information beyond the clinical model but do not establish a clinically meaningful improvement in decision-making. In particular, this study did not evaluate whether SII-guided assessment changes electrocardiographic monitoring, treatment decisions, or patient outcomes. Therefore, SII should be considered a candidate adjunct for further evaluation rather than an established tool for risk stratification or clinical decision-making.

Several limitations of this study should be acknowledged. First, this was a single-center retrospective study. Although bootstrap optimism correction was performed, the model has not been evaluated in an independent external cohort, and its transportability and clinical utility remain uncertain. Second, although the prespecified exclusion criteria helped define a more homogeneous analytic cohort and reduce potential confounding, a descriptive comparison showed that excluded patients were older and had a lower proportion of men than included patients; these differences may limit the generalizability of our findings to the broader AMI population (Supplementary Table S11). Third, SII was calculated from the earliest available presentation blood sample measured by the hospital laboratory, but the exact interval from symptom onset or admission to phlebotomy and its timing relative to reperfusion or other early treatments could not be reliably determined. Treatment before sampling may have affected the blood cell counts, and serial changes during hospitalization were not assessed. Fourth, despite expanded adjustment for available severity and treatment variables, residual confounding remains possible because electrolyte measurements and several other relevant structural, electrophysiological, and treatment-related factors were unavailable with sufficient completeness or consistent temporal alignment for analysis. Conversely, adjustment for variables downstream of AMI severity or inflammation may introduce overadjustment. Future multicenter studies with prospectively standardized data collection are warranted. Finally, MVA was identified from medical records; exact onset times were not consistently available for time-to-event analysis, precluding a reliable sensitivity analysis based on a 6 h cutoff after admission, and mild, transient, or incompletely documented arrhythmic episodes may not have been captured uniformly. Length-of-stay-stratified findings should also be interpreted cautiously because MVA may itself prolong hospitalization.

## 5 Conclusion

Higher SII remained associated with greater odds of in-hospital MVA after multivariable adjustment in patients with AMI, and the findings remained consistent across sensitivity analyses. Exploratory subgroup analyses suggested that the association varied across AMI phenotypes, although these findings require confirmation. As a simple biomarker derived from routine blood cell counts, SII showed modest apparent incremental prognostic information beyond conventional clinical variables in this cohort. Independent external validation is needed to establish its predictive performance and clinical utility before implementation.

## Data Availability

The raw data supporting the conclusions of this article will be made available by the authors, without undue reservation.
