# Supplementary material for: Association of the systemic immune-inflammation index with in-hospital malignant ventricular arrhythmia in patients with acute myocardial infarction
Source: Front Med (Lausanne). 2026 Oct 28;13:1939873. doi: 10.3389/fmed.2026.1939873 (PMC13752104; doi:10.3389/fmed.2026.1939873)
Supplement: Supplementary file 1 [file Supplementaryfile1.docx]

Supplementary Material

# 1 Supplementary Tables

**Supplementary Table S1. Missing data for covariates included in the multivariable models.**

| **Covariate** | **Missing, n** | **Missing, %** |
| --- | --- | --- |
| Age | 0 | 0 |
| Sex | 0 | 0 |
| Killip class | 366 | 2.72 |
| Heart rate | 37 | 0.27 |
| STEMI | 0 | 0 |
| Anterior myocardial infarction | 0 | 0 |
| Diabetes mellitus | 0 | 0 |
| Atrial fibrillation | 0 | 0 |
| History of coronary artery disease | 0 | 0 |
| History of stroke | 0 | 0 |
| LDL-C | 656 | 4.87 |
| Admission glucose | 86 | 0.64 |
| Albumin | 576 | 4.28 |
| Blood urea nitrogen | 34 | 0.25 |
| HbA1c | 861 | 6.39 |

Note: Missingness was assessed in the original dataset before multiple imputation (N = 13,465). No values were missing after MICE.

**Supplementary Table S2. Bootstrap optimism-corrected internal validation of model performance**

| **Performance measure** | **Clinical model, apparent** | **Clinical model, optimism-corrected** | **Clinical model + ln(SII), apparent** | **Clinical model + ln(SII), optimism-corrected** |
| --- | --- | --- | --- | --- |
| AUC | 0.797 | 0.788 | 0.814 | 0.806 |
| Calibration intercept | 0.000 | 0.002 | 0.000 | 0.002 |
| Calibration slope | 1.005 | 0.983 | 1.004 | 0.981 |
| Brier score | 0.0283 | 0.0286 | 0.0280 | 0.0283 |
| ΔAUC after adding ln(SII) | — | — | 0.017 | 0.018 |

Note: Optimism correction was based on 500 patient-level bootstrap samples, with identical resampling indices applied across the five imputed datasets. Both models were refitted in each bootstrap sample, and patient-level predicted probabilities were averaged across imputations. Calibration intercepts of 0 and slopes of 1 indicate ideal calibration, whereas lower Brier scores indicate better overall prediction accuracy.
Abbreviations: SII, systemic immune-inflammation index; AUC, area under the receiver operating characteristic curve.

**Supplementary Table S3. Apparent model discrimination across age, AMI type, and PCI status subgroups**

| **Subgroup** | **Patients, n** | **MVA events, n (%)** | **Clinical model AUC (95% CI)** | **Clinical model + ln(SII) AUC (95% CI)** | **ΔAUC** | **Holm-adjusted P value** |
| --- | --- | --- | --- | --- | --- | --- |
| **Age** |  |  |  |  |  |  |
| <65 years | 7,809 | 239 (3.1) | 0.790 (0.758–0.823) | 0.821 (0.791–0.851) | 0.031 | 0.002 |
| 65–74 years | 3,754 | 128 (3.4) | 0.825 (0.786–0.864) | 0.830 (0.790–0.869) | 0.005 | 1.000 |
| ≥75 years | 1,902 | 73 (3.8) | 0.771 (0.710–0.832) | 0.765 (0.702–0.828) | −0.006 | 1.000 |
| **AMI type** |  |  |  |  |  |  |
| STEMI | 2,175 | 93 (4.3) | 0.772 (0.719–0.825) | 0.756 (0.699–0.813) | −0.017 | 0.824 |
| NSTEMI | 11,290 | 347 (3.1) | 0.800 (0.774–0.826) | 0.825 (0.800–0.849) | 0.025 | <0.001 |
| **PCI status** |  |  |  |  |  |  |
| PCI | 9,690 | 294 (3.0) | 0.801 (0.773–0.829) | 0.824 (0.797–0.852) | 0.024 | 0.005 |
| No PCI | 3,775 | 146 (3.9) | 0.786 (0.744–0.828) | 0.788 (0.747–0.830) | 0.002 | 1.000 |

**Note:** Values represent apparent within-cohort discrimination. The clinical model included the prespecified Model 3 covariates, and the incremental model additionally included standardized ln(SII). Both models were fitted in the full cohort; patient-level predicted probabilities were averaged across the five imputed datasets and then evaluated within each subgroup without refitting. ΔAUC was calculated as the AUC for the model including ln(SII) minus that for the clinical model. P values were obtained using paired DeLong tests and adjusted across the seven subgroup comparisons using the Holm method. These analyses were exploratory and do not constitute external validation.
**Abbreviations:** SII, systemic immune-inflammation index; MVA, malignant ventricular arrhythmia; AUC, area under the receiver operating characteristic curve; CI, confidence interval; AMI, acute myocardial infarction; STEMI, ST-segment elevation myocardial infarction; NSTEMI, non-ST-segment elevation myocardial infarction; PCI, percutaneous coronary intervention.

Supplementary Table S4. Complete-case analysis for the association between SII and MVA

| **Category/unit** | **Events/N (%)** | **Model 0** | | **Model 1** | | **Model 2** | | **Model 3** | |
| --- | --- | --- | --- | --- | --- | --- | --- | --- | --- |
|  |  | **OR (95% CI)** | **P value** | **OR (95% CI)** | **P value** | **OR (95% CI)** | **P value** | **OR (95% CI)** | **P value** |
| Q1 | 33/2841 (1.2) | Reference |  | Reference |  | Reference |  | Reference |  |
| Q2 | 45/2891 (1.6) | 1.35 (0.86-2.11) | 0.198 | 1.35 (0.86-2.12) | 0.194 | 1.24 (0.78-1.96) | 0.364 | 1.26 (0.80-2.00) | 0.321 |
| Q3 | 67/2899 (2.3) | 2.01 (1.32-3.06) | 0.001 | 2.02 (1.33-3.08) | 0.001 | 1.79 (1.17-2.75) | 0.008 | 1.81 (1.17-2.78) | 0.007 |
| Q4 | 176/2925 (6.0) | 5.45 (3.74-7.93) | <0.001 | 5.47 (3.76-7.97) | <0.001 | 3.05 (2.05-4.53) | <0.001 | 3.04 (2.04-4.54) | <0.001 |
| **per-quartile increase** |  | 1.88 (1.67-2.11) | <0.001 | 1.88 (1.68-2.11) | <0.001 | 1.49 (1.32-1.68) | <0.001 | 1.48 (1.31-1.68) | <0.001 |
| Per 1-SD increase in ln(SII) | 321/11556 (2.8) | 1.97 (1.77-2.19) | <0.001 | 1.97 (1.77-2.19) | <0.001 | 1.49 (1.32-1.67) | <0.001 | 1.47 (1.31-1.65) | <0.001 |

Note: Odds ratios (ORs) and 95% confidence intervals (CIs) were estimated using logistic regression models.

Model 0 was unadjusted.

Model 1 was adjusted for age and sex.

Model 2 was further adjusted for Killip class (categorical), heart rate, STEMI, anterior myocardial infarction, diabetes mellitus, atrial fibrillation, history of coronary artery disease, and history of stroke.

Model 3 was further adjusted for LDL-C, admission glucose, albumin, blood urea nitrogen, and ln(HbA1c).

Abbreviations: SII, systemic immune-inflammation index; MVA, malignant ventricular arrhythmia; Q, quartile; SD, standard deviation; OR, odds ratio; CI, confidence interval; STEMI, ST-segment elevation myocardial infarction; LDL-C, low-density lipoprotein cholesterol; HbA1c, glycated hemoglobin.

SII quartiles were defined using the primary full-cohort cut-off values. This analysis used the original non-imputed dataset and included complete cases for SII, endpoint, and all model covariates.

Supplementary Table S5. Association between SII and MVA after additional adjustment for CK-MB and NT-proBNP

| **Category/unit** | **Events/N (%)** | **Model 0** | | **Model 1** | | **Model 2** | | **Model 3** | |
| --- | --- | --- | --- | --- | --- | --- | --- | --- | --- |
|  |  | **OR (95% CI)** | **P value** | **OR (95% CI)** | **P value** | **OR (95% CI)** | **P value** | **OR (95% CI)** | **P value** |
| Q1 | 46/3367 (1.4) | Reference |  | Reference |  | Reference |  | Reference |  |
| Q2 | 61/3366 (1.8) | 1.33 (0.91-1.96) | 0.145 | 1.34 (0.91-1.97) | 0.139 | 1.23 (0.83-1.82) | 0.302 | 1.28 (0.86-1.91) | 0.217 |
| Q3 | 98/3366 (2.9) | 2.16 (1.52-3.08) | <0.001 | 2.19 (1.53-3.11) | <0.001 | 1.88 (1.31-2.70) | <0.001 | 1.87 (1.29-2.71) | 0.001 |
| Q4 | 235/3366 (7.0) | 5.42 (3.94-7.46) | <0.001 | 5.47 (3.97-7.53) | <0.001 | 2.95 (2.10-4.14) | <0.001 | 2.74 (1.91-3.92) | <0.001 |
| **per-quartile increase** |  | 1.87 (1.69-2.06) | <0.001 | 1.87 (1.70-2.07) | <0.001 | 1.47 (1.33-1.63) | <0.001 | 1.42 (1.27-1.58) | <0.001 |
| Per 1-SD increase in ln(SII) | 440/13465 (3.3) | 2.00 (1.83-2.19) | <0.001 | 2.01 (1.84-2.20) | <0.001 | 1.50 (1.36-1.66) | <0.001 | 1.44 (1.30-1.60) | <0.001 |

Note: Odds ratios (ORs) and 95% confidence intervals (CIs) were estimated using logistic regression models.

Model 0 was unadjusted.

Model 1 was adjusted for age and sex.

Model 2 was further adjusted for Killip class (categorical), heart rate, STEMI, anterior myocardial infarction, diabetes mellitus, atrial fibrillation, history of coronary artery disease, and history of stroke.

Model 3 was further adjusted for LDL-C, admission glucose, albumin, blood urea nitrogen, ln(HbA1c), ln(CK-MB), and ln(NT-proBNP).

Abbreviations: SII, systemic immune-inflammation index; MVA, malignant ventricular arrhythmia; Q, quartile; SD, standard deviation; OR, odds ratio; CI, confidence interval; STEMI, ST-segment elevation myocardial infarction; LDL-C, low-density lipoprotein cholesterol; HbA1c, glycated hemoglobin; CK-MB, creatine kinase-MB; NT-proBNP, N-terminal pro-B-type natriuretic peptide.

SII quartiles were defined using the primary full-cohort cut-off values. In this sensitivity analysis, Model 3 was additionally adjusted for ln(CK-MB) and ln(NT-proBNP).

Supplementary Table S6. Association between SII and MVA after excluding patients with cardiogenic shock

| **Category/unit** | **Events/N (%)** | **Model 0** | | **Model 1** | | **Model 2** | | **Model 3** | |
| --- | --- | --- | --- | --- | --- | --- | --- | --- | --- |
|  |  | **OR (95% CI)** | **P value** | **OR (95% CI)** | **P value** | **OR (95% CI)** | **P value** | **OR (95% CI)** | **P value** |
| Q1 | 42/3343 (1.3) | Reference |  | Reference |  | Reference |  | Reference |  |
| Q2 | 56/3329 (1.7) | 1.34 (0.90-2.01) | 0.150 | 1.35 (0.90-2.02) | 0.147 | 1.27 (0.84-1.91) | 0.251 | 1.28 (0.85-1.92) | 0.246 |
| Q3 | 77/3323 (2.3) | 1.86 (1.28-2.72) | 0.001 | 1.87 (1.28-2.74) | 0.001 | 1.64 (1.12-2.42) | 0.012 | 1.64 (1.11-2.42) | 0.013 |
| Q4 | 180/3216 (5.6) | 4.66 (3.32-6.54) | <0.001 | 4.69 (3.34-6.59) | <0.001 | 2.94 (2.05-4.20) | <0.001 | 2.87 (2.00-4.13) | <0.001 |
| per-quartile increase |  | 1.75 (1.57-1.94) | <0.001 | 1.75 (1.58-1.95) | <0.001 | 1.46 (1.31-1.63) | <0.001 | 1.44 (1.29-1.62) | <0.001 |
| Per 1-SD increase in ln(SII) | 355/13211 (2.7) | 1.89 (1.71-2.09) | <0.001 | 1.90 (1.72-2.10) | <0.001 | 1.50 (1.35-1.67) | <0.001 | 1.47 (1.32-1.64) | <0.001 |

Note: Odds ratios (ORs) and 95% confidence intervals (CIs) were estimated using logistic regression models.

Model 0 was unadjusted.

Model 1 was adjusted for age and sex.

Model 2 was further adjusted for Killip class (categorical), heart rate, STEMI, anterior myocardial infarction, diabetes mellitus, atrial fibrillation, history of coronary artery disease, and history of stroke.

Model 3 was further adjusted for LDL-C, admission glucose, albumin, blood urea nitrogen, and ln(HbA1c).

Abbreviations: SII, systemic immune-inflammation index; MVA, malignant ventricular arrhythmia; Q, quartile; SD, standard deviation; OR, odds ratio; CI, confidence interval; STEMI, ST-segment elevation myocardial infarction; LDL-C, low-density lipoprotein cholesterol; HbA1c, glycated hemoglobin.

SII quartiles were defined using the primary full-cohort cut-off values. Patients with cardiogenic shock were excluded.

Supplementary Table S7. Association between SII and MVA after excluding patients with chronic kidney disease

| **Category/unit** | **Events/N (%)** | **Model 0** | | **Model 1** | | **Model 2** | | **Model 3** | |
| --- | --- | --- | --- | --- | --- | --- | --- | --- | --- |
|  |  | **OR (95% CI)** | **P value** | **OR (95% CI)** | **P value** | **OR (95% CI)** | **P value** | **OR (95% CI)** | **P value** |
| Q1 | 39/3092 (1.3) | Reference |  | Reference |  | Reference |  | Reference |  |
| Q2 | 53/3044 (1.7) | 1.39 (0.91-2.10) | 0.124 | 1.39 (0.92-2.11) | 0.119 | 1.31 (0.85-2.00) | 0.221 | 1.35 (0.88-2.07) | 0.174 |
| Q3 | 88/3057 (2.9) | 2.32 (1.59-3.39) | <0.001 | 2.34 (1.60-3.43) | <0.001 | 2.02 (1.36-2.98) | <0.001 | 2.01 (1.36-2.99) | <0.001 |
| Q4 | 213/3021 (7.1) | 5.94 (4.20-8.39) | <0.001 | 5.99 (4.24-8.46) | <0.001 | 3.26 (2.26-4.71) | <0.001 | 3.22 (2.22-4.67) | <0.001 |
| per-quartile increase |  | 1.93 (1.74-2.14) | <0.001 | 1.93 (1.74-2.15) | <0.001 | 1.52 (1.36-1.69) | <0.001 | 1.50 (1.34-1.68) | <0.001 |
| Per 1-SD increase in ln(SII) | 393/12214 (3.2) | 2.09 (1.90-2.29) | <0.001 | 2.10 (1.91-2.30) | <0.001 | 1.57 (1.41-1.74) | <0.001 | 1.54 (1.38-1.72) | <0.001 |

Note: Odds ratios (ORs) and 95% confidence intervals (CIs) were estimated using logistic regression models.

Model 0 was unadjusted.

Model 1 was adjusted for age and sex.

Model 2 was further adjusted for Killip class (categorical), heart rate, STEMI, anterior myocardial infarction, diabetes mellitus, atrial fibrillation, history of coronary artery disease, and history of stroke.

Model 3 was further adjusted for LDL-C, admission glucose, albumin, blood urea nitrogen, and ln(HbA1c).

Abbreviations: SII, systemic immune-inflammation index; MVA, malignant ventricular arrhythmia; Q, quartile; SD, standard deviation; OR, odds ratio; CI, confidence interval; STEMI, ST-segment elevation myocardial infarction; LDL-C, low-density lipoprotein cholesterol; HbA1c, glycated hemoglobin.

SII quartiles were defined using the primary full-cohort cut-off values. Patients with chronic kidney disease were excluded.

Supplementary Table S8. Association between SII and MVA after excluding patients with Killip class IV

| **Category/unit** | **Events/N (%)** | **Model 0** | | **Model 1** | | **Model 2** | | **Model 3** | |
| --- | --- | --- | --- | --- | --- | --- | --- | --- | --- |
|  |  | **OR (95% CI)** | **P value** | **OR (95% CI)** | **P value** | **OR (95% CI)** | **P value** | **OR (95% CI)** | **P value** |
| Q1 | 33/3310 (1.0) | Reference |  | Reference |  | Reference |  | Reference |  |
| Q2 | 49/3288 (1.5) | 1.50 (0.96-2.34) | 0.072 | 1.50 (0.96-2.34) | 0.072 | 1.41 (0.91-2.21) | 0.128 | 1.41 (0.90-2.20) | 0.135 |
| Q3 | 64/3274 (2.0) | 1.98 (1.30-3.02) | 0.002 | 1.99 (1.30-3.03) | 0.001 | 1.76 (1.15-2.70) | 0.009 | 1.75 (1.14-2.69) | 0.010 |
| Q4 | 138/3066 (4.5) | 4.68 (3.19-6.87) | <0.001 | 4.70 (3.20-6.90) | <0.001 | 3.51 (2.36-5.22) | <0.001 | 3.40 (2.27-5.08) | <0.001 |
| per-quartile increase |  | 1.71 (1.52-1.93) | <0.001 | 1.72 (1.53-1.93) | <0.001 | 1.54 (1.37-1.74) | <0.001 | 1.52 (1.34-1.72) | <0.001 |
| Per 1-SD increase in ln(SII) | 284/12938 (2.2) | 1.80 (1.61-2.02) | <0.001 | 1.81 (1.62-2.03) | <0.001 | 1.60 (1.42-1.80) | <0.001 | 1.56 (1.38-1.76) | <0.001 |

Note: Odds ratios (ORs) and 95% confidence intervals (CIs) were estimated using logistic regression models.

Model 0 was unadjusted.

Model 1 was adjusted for age and sex.

Model 2 was further adjusted for Killip class (categorical), heart rate, STEMI, anterior myocardial infarction, diabetes mellitus, atrial fibrillation, history of coronary artery disease, and history of stroke.

Model 3 was further adjusted for LDL-C, admission glucose, albumin, blood urea nitrogen, and ln(HbA1c).

Abbreviations: SII, systemic immune-inflammation index; MVA, malignant ventricular arrhythmia; Q, quartile; SD, standard deviation; OR, odds ratio; CI, confidence interval; STEMI, ST-segment elevation myocardial infarction; LDL-C, low-density lipoprotein cholesterol; HbA1c, glycated hemoglobin.

SII quartiles were defined using the primary full-cohort cut-off values. Patients with Killip class IV were excluded.

**Supplementary Table S9. Association between SII and MVA stratified by length of stay**

| Category/unit | Events/N (%) | Model 3 |  | P for interaction |
| --- | --- | --- | --- | --- |
|  |  | OR (95% CI) | P value |  |
| ≤3 days | 167/5324 (3.1) | 1.69 (1.42-2.01) | <0.001 | 0.001 |
| >3 to 7 days | 183/6069 (3.0) | 1.55 (1.32-1.81) | <0.001 |  |
| >7 days | 90/2072 (4.3) | 1.17 (0.94-1.46) | 0.154 |  |

Note: Odds ratios (ORs) and 95% confidence intervals (CIs) represent the association with MVA per 1-SD increase in ln-transformed SII within each length-of-stay stratum.
Model 3 was adjusted for age, sex, Killip class (categorical), heart rate, STEMI, anterior myocardial infarction, diabetes mellitus, atrial fibrillation, history of coronary artery disease, history of stroke, LDL-C, admission glucose, albumin, blood urea nitrogen, and ln(HbA1c).
The P value for interaction was obtained by including an interaction term between standardized ln-transformed SII and length-of-stay category.
Abbreviations: SII, systemic immune-inflammation index; MVA, malignant ventricular arrhythmia; SD, standard deviation; OR, odds ratio; CI, confidence interval; STEMI, ST-segment elevation myocardial infarction; LDL-C, low-density lipoprotein cholesterol; HbA1c, glycated hemoglobin.

**Supplementary Table S10. Association between SII and MVA restricted to documented sustained ventricular tachycardia or ventricular fibrillation**

| **Category/unit** | **Events/N (%)** | **Model 0** | | **Model 1** | | **Model 2** | | **Model 3** | |
| --- | --- | --- | --- | --- | --- | --- | --- | --- | --- |
|  |  | **OR**  **(95% CI)** | **P value** | **OR**  **(95% CI)** | **P value** | **OR**  **(95% CI)** | **P value** | **OR**  **(95% CI)** | **P value** |
| Q1 | 19/3367 (0.6) | Reference |  | Reference |  | Reference |  | Reference |  |
| Q2 | 26/3366 (0.8) | 1.37 (0.76–2.48) | 0.296 | 1.37 (0.75–2.47) | 0.303 | 1.28 (0.70–2.32) | 0.423 | 1.31 (0.72–2.38) | 0.379 |
| Q3 | 51/3366 (1.5) | 2.71 (1.60–4.60) | <0.001 | 2.70 (1.59–4.59) | <0.001 | 2.41 (1.41–4.11) | 0.001 | 2.45 (1.43–4.18) | 0.001 |
| Q4 | 128/3366 (3.8) | 6.97 (4.29–11.30) | <0.001 | 7.04 (4.33–11.42) | <0.001 | 4.18 (2.53–6.92) | <0.001 | 4.28 (2.58–7.10) | <0.001 |
| per-quartile increase |  | 2.07 (1.79–2.39) | <0.001 | 2.08 (1.80–2.41) | <0.001 | 1.68 (1.45–1.95) | <0.001 | 1.69 (1.45–1.96) | <0.001 |
| Per 1-SD increase in ln(SII) | 224/13465 (1.7) | 2.12 (1.88–2.39) | <0.001 | 2.16 (1.91–2.43) | <0.001 | 1.68 (1.47–1.92) | <0.001 | 1.69 (1.47–1.93) | <0.001 |

Note: The strict outcome comprised documented ventricular fibrillation or sustained ventricular tachycardia. For this alternative-outcome analysis, only patients meeting this strict definition were classified as events; all other cohort members were classified as non-events. Odds ratios (ORs) and 95% confidence intervals (CIs) were estimated using logistic regression models.
Model 0 was unadjusted.
Model 1 was adjusted for age and sex.
Model 2 was further adjusted for Killip class (categorical), heart rate, STEMI, anterior myocardial infarction, diabetes mellitus, atrial fibrillation, history of coronary artery disease, and history of stroke.
Model 3 was further adjusted for LDL-C, admission glucose, albumin, blood urea nitrogen, and ln(HbA1c).
Abbreviations: SII, systemic immune-inflammation index; MVA, malignant ventricular arrhythmia; VT, ventricular tachycardia; VF, ventricular fibrillation; Q, quartile; SD, standard deviation; OR, odds ratio; CI, confidence interval; STEMI, ST-segment elevation myocardial infarction; LDL-C, low-density lipoprotein cholesterol; HbA1c, glycated hemoglobin.
SII quartiles were defined using the primary full-cohort cut-off values.

**Supplementary Table S11. Demographic characteristics of included and excluded patients**

| **Characteristic** | **Included patients (n = 13,465)** | **Excluded patients (n = 1,414)** |
| --- | --- | --- |
| Age, years | 62 [53–70] | 66 [57–73] |
| Male sex, n (%) | 10,786 (80.1) | 1,067 (75.5) |

Note: Data are presented as median [interquartile range] or n (%). The comparison was descriptive, and no formal hypothesis testing was performed.

Abbreviation: IQR, interquartile range.
